# Supplementary material for: Cost–Benefit of Real-Time Multiplex PCR Testing of SARS-CoV-2 in German Hospitals
Source: Int J Environ Res Public Health. 2023 Feb 15;20(4):3447. doi: 10.3390/ijerph20043447 (PMC9960777; doi:10.3390/ijerph20043447)
Supplement: Supplementary file 1 [file ijerph-20-03447-s001.zip › ijerph-2059418-supplementary.pdf]

Cost–Benefit of Real-Time Multiplex PCR Testing of SARS-CoV-2 in German Hospitals

Online Supplement

1. Model input

1.1. Epidemiological and laboratory parameters

1.1.1. Prevalence of COVID-19 in hospital emergency rooms

Published data on the prevalence of COVID-19 in coming to German ER with symptoms compatible with COVID-19 and tested with a RT-PCR as reference are sparse. In *Teichgräber´*s prospective, single center cohort study [1], the diagnosis of COVID-10 was confirmed in 13/165 adults (7.9%) presenting with symptoms between 18 March and 28 April 2020. In the report of *Paul* et al. [2] comparing SARS-CoV-2-Antigen-Tests and a RT-PCR between 5 October and 7 November 2020, 41.2% of those symptomatic patients (42/102) who had to be hospitalized after visiting the ER (42/102) were COVID-19 positive, but no rate was reported for all patients visiting the ER. In an analysis of ER data in Germany of 475,067 patients in the first 22 calendar weeks in 2020 [3], a maximum of 15.6% COVID-19 positive test results (523/3353) was observed in calendar week 13.

As no more recent figures are available in the literature, we took this figure as baseline (95% CI 0.144 to 0.169) and choose a proportion of 41.2% as upper and 7.9% as lower bound values.

1.1.2. Sensitivity and specificity of clinical judgement

The clinical disease of COVID-19 is challenging due to its non-specific symptoms. The disease usually manifests itself as an infection of the respiratory tract, common symptoms are dry cough, fever, myalgia and dyspnea [4]. The only approximately pathognomonic symptom for COVID-19 is loss of smell and taste, which, however, occurs in around 22% of patients [5]. The often cited CT finding of bilateral, subpleurally imposing opacities of milk glass and consolidation of lung segments are not specific for COVID-19 and may also be present in other viral pneumonia [6].

As there are no gold criteria for a clinical case definition of SARS-CoV-2, widely varying values for sensitivity and specificity of diagnosing influenza on the basis of clinical symptoms alone have been reported:

Sensitivity and specificity of self-reported symptoms in English health care workers as an indication of COVID-19 infection were 74.5% (95% CI 71.3 to 77.6%) and 64.5% (95% CI 62.9 to 66.0%), respectively [7].

In *Xia*´s Chinese study [4], including 512 patients with COVID-19, a clinical sensitivity for detecting in COVID-19 diseases of 89.0% (95% CI 87.8 to 91.5%) was calculated and a specificity of 44.2% (95% CI.38.5 to 50.0%),

In another multicenter Chinese study, including a total of 905 symptomatic patients, *Mei* et al. [8] reported a sensitivity of 80.6% (95% CI 72.9 to 86.9%); and a specificity of 68.3% (95% CI 60.0 to 75.8%) when only clinical data (including laboratory results) were incorporated in their model.

In a Mexican cross-sectional study [9], analysing a cohort of 2173 patients, the symptomatological scale, constructed from 6 symptoms, resulted in a sensitivity of 83.5% and a specificity of 32.9% versus RT-PCT confirmed COVID-19.

In a German evaluation of a clinical COVID-19-Score [10], sensitivity was 98.4%, but specificity was only 48.3%.

We used the figures of *Mei* et al. [8] which showed the highest specificity as baseline.

1.1.3. Sensitivity and specificity of the Savanna® in symptomatic patients

According to the manufacturer´s specifications [11], the sensitivity of the Savanna® is 99.31% (95% CI 96.17 to 99.88) and the specificity is 100% (95% CI 95.42 to 100%), using the BioFire® Repiratory Panel 2.1 (Biomerieux) as reference method. Real-life studies in symptomatic patients from different countries are not yet available.

1.1.4. Rate of hospitalizations

According to the data of the survey “COVID-19-Fälle nach Meldewoche und Geschlecht sowie Anteile mit für COVID-19 relevanten Symptomen, Anteile Hospitalisierter und Verstorbener” of the German RKI, the rate of hospitalization in laboratory-confirmed COVID-19 cases in calendar weeks 10/2020 to 42/ 2022 was only 4.3% (723,862 hospitalized SARS-CoV-2 cases /16,781,36x cases with information on hospitalization) [12]. Although this figure importantly indicates a downward trend, it does not establish a causal relationship between COVID-19 test positivity and the necessity of hospitalization due to disease severity. According to the results of the German ReCovER register on inpatient admission in six German university ER [13], 50% of the laboratory confirmed SARS-CoV-2 cases (297/594)

were subsequently admitted to normal stations and 10.4% to intensive care units (62/594). Accordingly, we used the figure of 60.44% (359/594) as base-case estimate for all COVID-19 suspects and took the upper (64.29%) and the lower bound (56.45%) of the 95% confidence interval in univariate sensitivity analysis. For probabilistic sensitivity analysis (PSA), however, we decided to use the formal hospitalization rate of 4.3% currently reported by the RKI as lower bound in order to cover the widest possible range of situations.

1.1.5. Secondary attack rate after COVID-19 transmission in healthcare workers (HCW)

Data on the secondary attack rate of COVID-19, defined as near contact with new onset of acute respiratory COVID-19 illness, are sparse. Since 15 March 2022, vaccination for all HCW in German hospitals been a requirement.; Thanks to vaccination prioritization, however, 94 percent of physicians and 90 percent of nurses in Germany had already been fully vaccinated by July 2021 [14].

In a in a recently published analysis of a German outbreak the instantaneous, real-life risk of SARS-CoV-2 transmission from an infective index case to a fully-vaccinated HCW contact leading to symptoms was 2.48% (3/121; 95% CI 0.85% to 7.4%) [15]. We took this figure as base-case value.

1.1.6 Duration of hospital stay

To date, data on the average duration of an uncomplicated COVID-19 disease in employed persons in Germany have not yet been published. Accordingly we utilized the median length of hospital stay of 10 days (IQR 5-19) published for all COVID-19 patients by *Tolksdorf* [16], taking the lower (25 th) percentile as minimum duration of 5 days and the 75th percentile as upper bound.

1.2 Economic parameters

1.2.1. Costs of the Savanna® Respiratoy Viral Panel-4

According to the information provided by Quidel Inc. the costs of the Savanna® are uniformly €40 (inclusive user fee).

1.2.2. Reimbursement of PCR-testing in ambulatory patients who will be discharged again

Costs of Savanna testing in the ER in Covid-19 patients who can be discharged home due to lack of disease severity may be partially compensated with € 30 according to § 9 Satz 2 TestV [17]. Thus, a deficit of €10 per test remains at the expense of the hospital.

1.2.3 Reimbursement of PCR-testing in inpatients

A PCR for respiratory pathogens, and thus also for detection of COVID-19, may be charged according to the positions 4780 Isolation of nucleic acids, €52.46), 4782 (enzymatic transcription of RNA using reverse transcriptase, €29.14), 4783 (amplification of nucleic acids or nucleic acid fragments using the polymerase chain reaction (PCR), € 29.14) and 4785 (identification of nucleic acid fragments by hybridization with radioactively or non-radioactively labeled probes and subsequent detection, per probe € 17.49) of German Scale of Medical fees (GOÄ) that regulates the billing of medical services outside the statutory medical care in Germany, summing up to a total of €128.23 (without application of the standard increase rate of 1.15). However, taking into account on the competition between laboratories, significant discounts on official fee positions are agreed between external laboratory and the respective hospital. Accordingly, as base-case value for our model, we considered the price of €37.80 which will be reimbursed by the statutory or private health insurances to the hospital since 1.7.2022 according to the 3rd agreement pursuant to Section 26(2) of the Hospital Act for testing for SARS-CoV-2 in the hospital [18]. However, as the agreements between external laboratories and hospitals may vary and published data do not exist, we estimate the price that laboratories actually charge vis-à-vis hospitals to be up to 50% higher (€56.70) than the base-case. We took this figure as upper bound in sensitivity analysis

In contrast, the current price of one Savanna® test (cassette and fee for using the device) is €40. Therefore, by using the Savanna® in the ER, the difference of €2.8 per test to the remunerated €37.8 is borne by the hospital itself.

1.2.3. Hospital opportunity costs

Under the premise that most patients are accommodated in a twin-bedded room and that hospital wards in Germany are working at full capacity, the loss of the use of one bed is incurred by the hospital during the initial isolation period until the report of a PCR test has been arrived on the following day. In case that a PDR test has already been

reported at the same day of hospitalization. New patients who could have been placed in the blocked beds are turned away. Consequently, the hospital will forego revenue (incur opportunity costs). These costs are not covered by healthcare insurance. To arrive at a representative estimate of the cost to hospitals of an unused bed, the minimal average revenue per bed and day was determined on the basis of the G-DRG catalog (version 2022, InEK GmbH). Using the DRG for COVID-19 patients with viral pneumonia at the time of admission (G-DRG E79 C, respiratory infection), who would have occupied a second, but now blocked bed in the twin-bedded room, as follows:
The reimbursement per bed for one day is calculated as follows:
As described in [19], the cost weight for G-DRG E79C (based on ICD-10 codes J12.8 [Pneumonia form other pathogens], Z11 [Special screening examination for other viral diseases] and U99.0! [Special procedures for testing for SARS-CoV-2]) is 0.593. A discount of 0.384 must be deducted due to falling below the lower limit of 2 days of hospital stay. The remaining cost weight of 0.209 is then multiplied by €3,833.07 (the German base rate in 2022). The reimbursement for one day nursing (€148.72) has to be added to the subtotal of €801.11, resulting in €979.37
Since the hospital also saves costs (variable costs) by the non-treatment of a new case, the amount of revenue loss was reduced by 25% as suggested in hospital management theory [20] resulting in a value of €734.53 of opportunity costs for the unnecessarily blocked bed. When taking the clinical approach without internal RT-PCR testing, opportunity costs fall away if a negative PCR result of the swab of a COVD-19 suspect is reported by the external laboratory to the hospital on the next subsequent day.

1.2.4. Revenue by POC antigen testing before discharge
Early in-house testing with a RT-PCR 14 days after the onset of symptoms before discharged from hospital may reduce at least one day the hospital stay of an COVID-19 patient, although the DRG flat reimbursement rate per COVID-19 case stays the same. Additional revenue of in favor of the hospital will be achieved by dividing the total DRG-revenue (except for nursing) for a stay of COVID-19 patients (ICD-10 J12.8 in combination with U07.1!) of 6.9 days, €2,273.01 that the hospitals earns for the patient by 6.9, i.e., €329.42 given that the patient will be treated in hospital for at least 2 days (minimum length of stay for a DRG service). Thus, under the assumption that a patient can be discharged one day earlier due to the negative result of a POC antigen

test (rather than waiting for the negative result or a positive PCR result below a defined threshold value indicating a low patient´s viral load, the hospital saves €329.42.

1.2.5. Cost of intensified thromboembolism prophylaxis and dexamethasone

The German guidelines for hospitalized patients with COVID-19 [21] recommend intensified thromboembolism prophylaxis. If the external laboratory PCR result is negative, the anticoagulant prophylaxis may be discontinued. The recommended halfe-therapeutical dose, e.g. of enoxaparin for a patient weighting 80 kg is 8.000 IE (80 mg)/0.8 ml per day. The price of 24 ready-to-fill syringes a 8000 IE is €165.95, that of 1 syringe is €6.91; enoxaparin is considered to be administered once daily. Patients with COVID-19 who require low-flow/high-flow oxygen therapy or non-invasive/invasive ventilation, systemic corticosteroids should be given. Therapy should be initiated with 6 mg dexamethasone p.o. or i.v. for ten days. The lowest price in Germany of 50 tablets dexamethasone a 4 mg, is €45.24, so that the cost of 6 mg (1.5 tablets) is €1.36 per day (Rote Liste® 2022).

1.2.6. Costs of intrahospital transmission to HCW

In cases of unidentified COVID-19, the hazard of COVID-19–transmission to health care workers (HCW) as contacts of an initially unidentified COVID-19 patient must be considered. In Germany, employees receive full salary during sick leave. To date, data on the average duration of an uncomplicated COVID-19 disease in German employed persons have not yet been published.

Rommel et al. use an estimate of 15 days for Germany [22] whilst, Jo et al. [23] estimate a duration of 27.4 days for Korea. Accordingly, we assumed a minimum duration of 15 days with an upper bound of 27 days. Personnel costs were based on average rates obtained from the most recent data of the German Federal Statistical Office [24]: In 2021 the gross annual earning of a hospital employee was €62.377. Divided by 365 days the loss for the hospital as employer is €170.90 (rounded) per day.

References:

1. Teichgräber, U.; Malouhi, A.; Ingwersen, M.; Neumann, R.; Reljic, M.; Deinhardt-Emmer, S.; Löffler, B.; Behringer, W.; Lewejohann, J.-C.; Stallmach, A.; et al. Ruling out COVID-19 by chest CT at emergency admission when prevalence is low: the prospective, observational SCOUT study. *Respir. Res.* **2021**, *22*, 1–11, https://doi.org/10.1186/s12931-020-01611-w.
2. Paul, G.; Plecko, T.; Sethi, S.; Schilling, T.; Wienand, O.; Jürgensen, J. S.; Menzel, C. U. Klinische Performance eines neuen SARS-CoV-2-Antigen-Tests in der Notaufnahme eines Maximalversorgers. *Epid. Bull.* **2021**, *3*, 10-15.
3. Slagman, A.; Behringer, W.; Greiner, F.; Klein, M.; Weismann, D.; Erdmann, B.; Pigorsch, M.; Möckel, M.; AKTIN Emergency Department Registry; German Forum of University Emergency Departments (FUN) in the Society of University Clinics of Germany E.V. Medical Emergencies during the COVID-19 Pandemic — an Analysis of Emergency Department Data in Germany. *Dtsch. Arztebl. Int.* **2020**, *117*, 545-552.
4. Xia, Y.; Chen, W.; Ren, H.; Zhao, J.; Wang, L.; Jin, R.; Zhou, J.; Wang, Q.; Yan, F.; Zhang, B.; et al. A rapid screening classifier for diagnosing COVID-19. *Int. J. Biol. Sci.* **2021**, *17*, 539–548, https://doi.org/10.7150/ijbs.53982.
5. Robert Koch Institut. SARS-CoV-2 Steckbrief zur Coronavirus-Krankheit-2019 (COVID-19) 2020. Available online: https://www.rki.de/DE/Content/InfAZ/N/Neuartiges_Coronavirus/Steckbrief.html (accessed on 6 October 2020).
6. Auvinen, R.; Nohynek, H.; Syrjänen, R.; Ollgren, J.; Kerttula, T.; Mäntyläet, J.; Ikonen, N.; Loginov, R.; Haveri, A.; Kurkela, S.; Skogberg, K. Comparison of the Clinical Characteristics and Outcomes of Hospitalized Adult COVID-19 and Influenza Patients – a Prospective Observational Study. *Infect. Dis.* **2021**, *53*, 111-121.
7. Shorten, R.J.; Haslam, S.; Hurley, M.A.; Rowbottom, A.; Myers, M.; Wilkinson, P.; Orr, D. Seroprevalence of SARS-CoV-2 Infection in Healthcare Workers in a Large Teaching Hospital in the North West of England: A Period Prevalence Survey. *BMJ Open.* **2021**, *11*, e045384, https://doi.org/10.1136/bmjopen-2020-045384.
8. Mei, X.; Lee, H.-C.; Diao, K.-Y.; Huang, M.; Lin, B.; Liu, C.; Xie, Z.; Ma, Y.; Robson, P.M.; Chung, M.; et al. Artificial intelligence–enabled rapid diagnosis of patients with COVID-19. *Nat. Med.* **2020**, *26*, 1224–1228, https://doi.org/10.1038/s41591-020-0931-3.
9. Romero-Gameros, C.A.; Colin-Martínez, T.; Waizel-Haiat, S.; Vargas-Ortega, G.; Ferat-Osorio, E.; Guerrero-Paz, J.A.; Intriago-Alor, M.; López-Moreno, M.A.; Cuevas-García, C.F.; Mendoza-Zubieta, V.; et al. Diagnostic accuracy of symptoms as a diagnostic tool for SARS-CoV 2 infection: a cross-sectional study in a cohort of 2,173 patients. *BMC Infect. Dis.* **2021**, *21*, 1–11, https://doi.org/10.1186/s12879-021-05930-1.
10. Hüfner, A.; Kiefl, D.; Baacke, M.; Zöllner, R.; Mencía, E.L.; Schellein, O.; Avan, N.; Pemmerl, S. Risikostratifizierung durch Implementierung und Evaluation eines COVID-19-Scores. *Med. Klin. - Intensiv. und Notfallmedizin* **2020**, *115*, 132–138, https://doi.org/10.1007/s00063-020-00754-4.

11. Quidel. Available online: https://www.quidel.com/immunoassays/rapid-sars-tests/Savanna-sars-antigen-fia (accessed on 10 November 2022).
12. Robert Koch Institut. COVID-19-Fälle nach Meldewoche und Geschlecht sowie Anteile mit für COVID-19 relevanten Symptomen, Anteile Hospitalisierter/Verstorbener und Altersmittelwert/-median (Tabelle wird jeden Donnerstag aktualisiert). Available online: https://www.rki.de/DE/Content/InfAZ/N/Neuartiges_Coronavirus/Daten/Klinische_Aspekte.html (accessed on 05 November 2022).
13. Suárez, V.; Koehler, F.C.; Hackl, M.J.; Möckel, M.; Slagman, A.; Pudasaini, S.; Risse, J.; Schunk, D.; Blaschke, S.; Kümpers, P.; et al. Universitäre Notaufnahmen in der Coronapandemie – Ergebnisse des ReCovER-Registers. Med. Klin. - Intensiv. und Notfallmedizin 2021, 117, 558–567, https://doi.org/10.1007/s00063-021-00859-4.
14. Robert Koch Institut. KROCO – Krankenhausbasierte Online-Befragung zur COVID-19-Impfung. Available online: https://www.rki.de/DE/Content/InfAZ/N/Neuartiges_Coronavirus/Projekte_RKI/KROCO.html (accessed on 10 November 2022).
15. Robert Koch Institut. Untersuchung und Eindämmung eines SARS-CoV-2-Alpha-Ausbruchs in einer Pflegeeinrichtung im Landkreis Dithmarschen, Juni 2021. Available online: https://edoc.rki.de/handle/176904/10372 (accessed on 10 November 2022).
16. Tolksdorf, K.; Buda, S.; Schuler, E.; Wieler, L. H.; Haas, W. Eine höhere Letalität und lange Beatmungsdauer unterscheiden COVID-19 von schwer verlaufenden Atemwegsinfektionen in Grippewellen. *Epid. Bull.* **2020**, *41*, 3-10.
17. Coronavirus-Testverordnung vom 21. September 2021. Available online: https://www.bundesanzeiger.de/pub/publication/wXbxqfkUdLM85aXaEg9/content/wXbxqfkUdLM85aXaEg9/BAnz%20AT%2024.11.2022%20V2.pdf?inline (accessed on 10 November 2022).
18. Dritte Vereinbarung nach § 26 Absatz 2 Krankenhausgesetz über ein Zusatzentgelt für Testungen auf das Coronavirus SARS-CoV-2 im Krankenhaus. Available online: https://www.dkgev.de/themen/finanzierung-leistungskataloge/covid-19/vereinbarung-nach-26-abs-2-khg/ (accessed on 10 November 2022).
19. Multiple Choices. Available online: https://www.drg-research-group.de/index.php?option=com_webgrouper&Itemid=112&view=webgrouper (accessed on 07 November 2022).
20. Haubrock, M. Krankenhausfinanzwirtschaft. In *Betriebswirtschaft und Management im Krankenhaus*; Haubrock, M., Schär, W., Eds.; Huber: Bern, Switzerland, 2007; pp. 394–453.
21. S3-Leitlinie - Empfehlungen zur stationären Therapie von Patienten mit COVID-19. Available online: https://register.awmf.org/assets/guidelines/113-001LGl_S3_Empfehlungen-zur-stationaeren-Therapie-von-Patienten-mit-COVID-19_2022-09_1.pdf (accessed on 10 November 2022).
22. Rommel, A.; von der Lippe, E.; Plass, D.; Ziese, T.; Diercke, M.; der Heiden, M.A.; Haller, S.; Wengler, A.; BURDEN 2020 Study Group. The COVID-19 Disease Burden in Germany in 2020—Years of Life Lost to Death and Disease Over the Course of the Pandemic. *Dtsch. Arzteblatt Int.* **2021**, *118*, 145–151.

23. Jo, M.-W.; Go, D.-S.; Kim, R.; Lee, S.W.; Ock, M.; Kim, Y.-E.; Oh, I.-H.; Yoon, S.-J.; Park, H. The Burden of Disease due to COVID-19 in Korea Using Disability-Adjusted Life Years. *J. Korean Med Sci.* **2020**, *35*, e199, https://doi.org/10.3346/jkms.2020.35.e199.
24. Federal Statistical Office of Germany (Statistisches Bundesamt, Wiesbaden) - ESTATIS. Fachserie 16, Reihe 2.3: Verdienste und Arbeitskosten –Arbeitnehmerverdienste. 4.5.1 Durchschnittliche Bruttojahresverdienste und Sonderzahlungen nach Wirtschaftszweigen Arbeitnehmer im Jahr 2021 im Krankenhaus (Q861 Krankenhäuser). Available online: https://www.destatis.de/DE/Service/Bibliothek/_publikationen-fachserienliste-16.html#605986 (accessed on 10 November 2022).
